# A critical insight into the development, regulation and future prospects of biofuels in Canada

**DOI:** 10.1080/21655979.2021.1996017

**Published:** 2021-12-02

**Authors:** Rahul Saini, Carlos Saul Osorio-Gonzalez, Satinder Kaur Brar, Raymond Kwong

**Affiliations:** a Department of Civil Engineering, Lassonde School of Engineering, York University, North York, Canada; b Department of Biology, York University, North York, Canada

**Keywords:** Biofuel, blending mandates, GHG mitigation, clean fuel standards, scale-up

## Abstract

Renewable biofuel has a great potential in replacing the conventional transportation fuels as well as aiding the current issue of climate change and global warming. In the present scenario, tremendous initiatives have been implemented to encourage large-scale biofuel production and reduce greenhouse gas emissions. However, the information on the current biofuel status specifically in Canada and where it lacks in biofuel production, tax rebate and policies in comparison with other countries is limited. In this sense, the current work focuses on the liquid biofuel status, recent advancements and evaluation of programs aimed at reducing greenhouse gas emissions in coming years. Additionally, the role of private and government programs in scaling up the projects is elaborated using several examples of successful as well as failed attempts to commercialize biofuels. Moreover, the Canadian government regulations and policies for greenhouse gas mitigation, and biofuel blending policies are also briefly described. In summary, future aspects and suggestions to further increase biofuel production are portrayed in this review.

## 1. Introduction

Renewable biofuel has received considerable attention due to its ability to replace fossil fuel as well as meet growing energy demand. Several countries, such as the USA, Canada, United Kingdom and France, have developed numerous policies to decrease the usage of fossil fuel-based energy, reduce greenhouse gas emissions and promote biofuel production from renewable sources such as lignocellulosic biomass, energy crops, as well as domestic and industrial wastes [1]. As per the sustainable developmental goal report, it has been estimated that more than 700 policies have been implemented. Over 83 countries around the world have set up the 10-year framework on sustainable production and consumption strategies. For instance, under renewable energy directives, European Union aims to reduce greenhouse gas emission by 55% and increase 32% in renewable targets [2]. Likewise, International Energy Agency (IEA) has launched the ‘Methane Tracker 2020’ initiative to track the total methane emissions from oil and gas operations as they are the second-largest sector contributing to global warming. A slight rise in methane emissions was seen in 2019, however, due to the coronavirus (COVID-19) pandemic, a great deal of uncertainty regarding the future of energy consumption has risen [3]. Moreover, the current fall in methane gas or carbon dioxide emission due to less oil or gas consumption should not be taken for granted. For instance, a decrease in oil and gas operations revenue could mean that industries might reduce their attention in tackling methane gas emissions. Additionally, methane gas could be used as biofuel too. Hence, recycling the gas as energy could help in reducing the GHG emissions. Nevertheless, robust policies and initiatives should be taken into consideration to tackle this worldwide problem.

**Figure 1.**
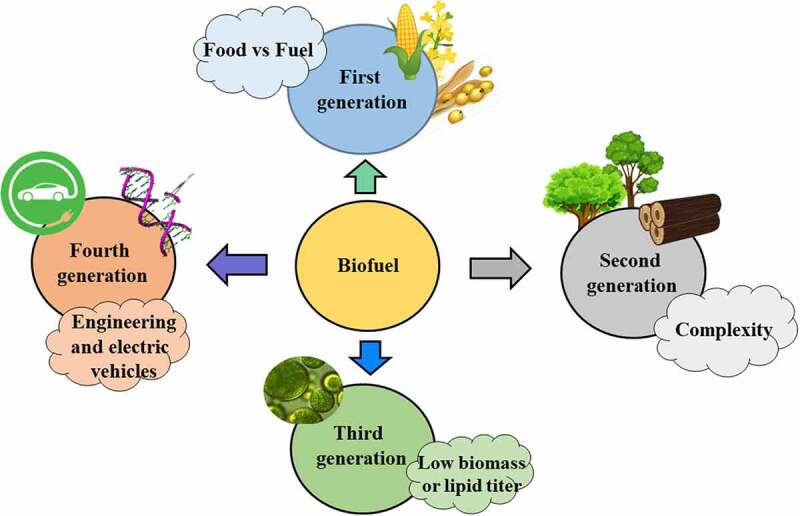
Displays the generation of biofuels as well as associated challenges with each generation

In general, biofuel production has been subject to constant transformation, for instance, reduction in biofuel production cost, utilization of renewable feedstocks, and process improvement to obtain maximum biofuel yield are being prioritized. In general, different types of feedstocks can be used for biofuel production, which has been further divided into different biofuel generations. Figure 1 illustrates the different generations of biofuels and the problems associated with each generation. For instance, first-generation biofuels are made using starch or sugar-based feedstocks as well as from vegetable oils [4]. Nevertheless, first-generation biofuels struggle to meet the low carbon fuel standards (LCFS) policy due to the energy involved in crop production, natural gas usage for transportation, and plant operations. The second generation of biofuel production includes the use of non-food feedstock, such as forestry residues, agricultural residues, and industrial residues. Although, it addressed the food versus fuel dilemma, but involves the use of complex feedstocks such as lignocellulosic biomass, resulting in the requirement of more energy and chemicals, as well as, more processing steps to transform feedstock into compatible biofuels [5]. On the other hand, third-generation biofuels include algae-based biofuel production [6], while fourth-generation biofuel includes genetic and metabolic engineering of microorganisms to increase their growth and lipid accumulation [7]. Of the four generations, only the first- and second-generation biofuels have been commercialized. However, the large-scale production of third- and fourth-generation biofuel is surrounded by several complications such as lower biomass production, substrate availability, high upstream and downstream cost as well as potential health and environmental concerns [7]. Nevertheless, the commercialization of biofuels is determined by several factors including their sustainability, production titer, environmental impacts, subsidies and funding availability. In particular, techno-economic analysis and life cycle assessment are commonly used to identify the economic feasibility and the potential environmental impact of the biofuels and the chemical used in their production [8].

In this sense, the article involves the currently active projects and critically analyses the type of feedstocks used. The current challenges in the scale-up of biofuel production through illustrative case studies of project scale-ups have been discussed. Also, the Canadian government regulations and policies for reducing the greenhouse gas (GHG) emissions and expanding biofuel market have been compared with leading biofuel producing countries and deliberated briefly. Additionally, possible economic conflicts in biofuel production have also been explained. In summary, future aspects of biofuel, the role of funding, and suggestions to further increase biofuel production are described in detail.

## 2. Current biofuel status and commercially active projects

**Figure 2.**
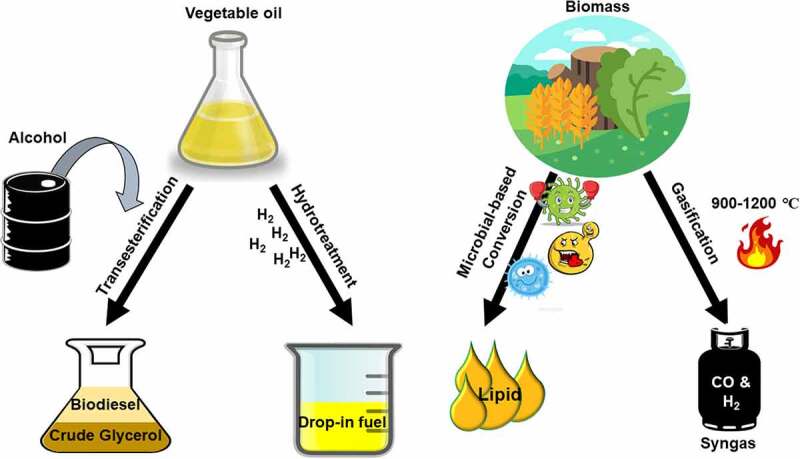
Illustration of different types of biofuels producing techniques

Over the past decade, several articles have been published explaining the types of biofuel generations, procedures, precautions, and technologies to increase biofuel production, including utilization of native microbial strains, metabolic engineering, optimization of fermentation processes, and consumption of renewable substrates [9–12]. In general, biofuel production falls into four categories: (i) transesterification of fatty acids from vegetable oils and free fatty acids; (ii) hydrotreatment of animal fats and vegetable oils; (iii) microbial-based biomass conversion or carbon-containing sources such as lignocellulosic biomass, domestic waste or industrial waste; and (iv) thermochemical conversion of lignocellulosic biomass into syngas [13]. Figure 2 illustrates the different procedures of renewable biofuel production.

Nevertheless, despite several successful laboratory reports, only a few projects on biofuel (including biodiesel, bioethanol and drop-in fuel) production have been commercialized [14]. For instance, for biodiesel, plants including ADM-Lloydminster (320 million liters per year), Consolidated Biofuels (11 million liters per year), and Verbio-Welland (170 million liters per year) are active in Canada. Table 1 illustrates different biofuel (biodiesel and bioethanol) producing industries in Canada. In general, Canada has a total of 14 ethanol plants and 6 biodiesel plants with a total capacity of 2145 and 629 million liters per year, respectively. Similarly, plants such as Greenfield, Enerkem, Eni are commercially active for large-scale bioethanol production. Likewise, La Mede, a France-based biorefinery, is one of the industries known to produce 500 kilo-tonnes of drop-in fuel per year using feedstock such as vegetable oils, animal fats, used oils, and residual fatty acids. In addition, an example of the implementation of engineered microorganisms for biofuel production and its commercialization is Gevo Inc. They successfully produce isobutanol in genetically engineered yeast using corn as a substrate, which is being transformed into sustainable aviation fuels for commercialization. Regardless, 85–90% of the current biofuel producing industries rely on vegetable oils and cereals because of their facile, cheaper and faster conversion into biofuels than other sources such as lignocellulosic biomass [5]. Nonetheless, the increasing population will put immense pressure on food industries hence, it would not be a viable option for biofuel production using vegetable oil or cereals in near future.

**Table 1.** List of biofuels producing industries in Canada

Company	Location	Feedstock	Capacity (Million Liters per Year)	Website
Biodiesel				
ADM	Lloydminster, Alberta	Canola oil	320	https://www.adm.com/
Verbio	Welland, Quebec	Canola and soybean oil	170	https://www.verbio.us/
Darling Ingredients	Montreal, Quebec	Animal fats and cooking oil	56	https://www.darlingii.com/
Consolidated biofuels Ltd.	Delta, British Colombia	Beef tallow and restaurant grease	11	https://consolidatedbiofuels.net/
Bioethanol				
Suncor Energy	St. Clair, Ontario	Corn	400	https://www.suncor.com/en-ca
IGPC	Chatham, Ontario	Corn	380	https://www.igpc.ca/
Greenfield	Johnstown, Ontario	Corn	260	https://greenfield.com/
Greenfield	Varennes. Quebec	Corn	190	https://greenfield.com/
Husky	Lloydminster, Saskatchewan	Cereals	150	https://huskyenergy.com/
Kawartha Ethanol Inc.	Havelock, Ontario	Corn	110	https://kawarthaethanol.ca/
Permolex	Red deer, Alberta	Cereals	45	https://permolex.com/

As a result, researchers are focusing on alternatives such as the microbial-based conversion of industrial, agricultural or domestic wastes into biofuels. Moreover, these industries are in constant backlog due to low microbial biomass yield, requirement of pretreatment methods, higher upstream/downstream cost and limited biofuel titer [5]. In addition, commercialization of biofuel production is also dependent on funds or subsidies, biofuel properties/purification, market standards and constant competition with low market-priced gasoline and diesel. Hence, it further requires exploration and deeper understanding to increase biofuel production and reduce production cost. Nevertheless, the government policies and regulatory norms play a crucial factor in increasing the biofuel production as well as laying down the initiatives to replace the conventional fuels through funding programs, tax rebate and advertisements explaining the benefits of biofuel usage.

## 3. Biofuel related regulations in Canada

Over the past decade, global oil demand has been increasing exponentially than the production rate. In Canada alone, 3000 million liters of ethanol were consumed in 2018, while 1700 million liters of ethanol were produced, and more than 1300 million liters were imported to meet the ever-increasing fuel demand. Likewise, biomass-based diesel consumption has increased from 500 million liters in 2015 to 800 million liters in 2018, while biodiesel production increased by 25% only due to lack of infrastructure and low production rate, resulting in an increased biofuel import and the associated flow of revenue outside the country.

Moreover, in 2017, the methane emissions from the oil sector producing petroleum have reached 2.4 billion tonnes of CO_2_ equivalent, which further contributed to global warming [3]. Hence, under the Clean Fuel Standard (CFS) initiative, the Canadian government aims to reduce GHG emissions by 30 million tonnes, and thereby, contributing to a 30% reduction in overall GHG emissions by 2030 [15]. Additionally, Advanced Biofuel Canada (ABC) and Renewable Industries Canada (RIC), in collaboration, conducted a study in 2019 about the CFS policy and demonstrated that the use of biofuels could help reduce GHG emission as much as 21.3 megatons/year by the end of 2030, which would be 70% of the 30 million tonne reduction goal of the federal government [16]. Under the CFS policy, Canada targets to increase the biofuel production capacity from 3 to 8.5 billion liters per year by 2030. However, it has been estimated that enhancing the use of biofuel from 7% to 15% by 2030 would decrease GHG emissions by 14 million tonnes per year [17]. Therefore, despite the provincial and federal-level actions, Canada is still far behind to meet its target in 2030.

To tackle this situation and limit the use of petroleum-based fuels, the Canadian government has mandated the renewable content in diesel and gasoline to be not less than 2% and 5%, respectively, while several provinces within the country have further improvised the blend requirement to increase the biofuel production and decrease the carbon emission. For instance, Manitoba and Saskatchewan mandate bioethanol in gasoline blends of 8.5% and 7.5%, respectively, while both Ontario and British Columbia have mandated a requirement of 4% biodiesel blend [17]. Moreover, as per the report by Renewable Industries Canada, Ontario is increasing the ethanol blend in gasoline to 15% by 2030, which would ultimately aid in increasing bioethanol production [18]. Also, Renewable Industry Canada has launched a new information campaign to promote the low-carbon fuel standard (LCFS) and to decrease the carbon print in the transportation sector ultimately aiding in reducing the GHG emission in the environment [19]. This would promote new economic investments to further improve biofuel production, expand the business, decrease imports and improve efficiency.

Besides, biofuel production is in a competitive backlog for scaling-up and commercialization in Canada. This can be attributed to the exponential increase in shale gas production in North America, which has resulted in a drastic reduction of prices for both natural gas and LPG. Moreover, another reason biofuel lags is due to their low energy density than conventional fuels. For instance, bioethanol has 33% less energy density than gasoline, i.e., a greater volume of bioethanol would be consumed to drive the same distance, compared to gasoline [20]. This would, consequently, mean that consumers will pay extra excise taxes due to the consumption of a greater volume of biofuel. The lower the energy density of biofuel, the higher will be the taxes, for instance, the tax for federal gasoline is $2.88/gigajoules, while that for gasoline with 6% ethanol is $2.94/gigajoules. In contrast, fuel vehicles that run on natural gas have paid less tax per kilometer [20].

Nonetheless, despite numerous industrial and government initiatives, the emissions associated with fossil fuels are still high. Compliance and wider acceptance of the policies developed toward sustainable development would be critical to achieving 75% greenhouse gas (GHG) reduction by the end of 2030. Hence, the CFS program should recognize funding entities and develop fair tax policies and standards to attract capital investments from the private sector to get aid in developing commercial fuel production capacity. The following suggestion could be considered for policy reformation. Firstly, increasing the bioethanol blending to 85% as well as adapting sustainable aviation fuel will encourage the industries to increase biofuel production and reduce greenhouse gas emissions by replacing conventional fuels. Secondly, fast action on life cycle assessments models on the environment and climate change will allow us to make necessary amendments in a timely manner. Thirdly, strict timeline should be put in place for regulation publications and their timely implementation. Finally, increasing the use of renewable and sustainable substrates for biofuel production will help ease the pressure on Canadian farm practices.

## 4. Economic investments and challenges

There is no denying in the fact that economics plays a crucial role in the development and commercialization of biorefineries. In general, the governmental policies and regulations govern the footing of industries in the market, which further varies with countries, consumers and market standards. Numerous government programs are currently funding several projects to scale up the biofuel production, cover the capital cost, and provide start-up loans. Billions of investments have been made throughout the world. Table 2 displays the different biofuels policies laid in different parts of continents by their respective governments. For instance, the Canadian government has completed two biofuel funding programs, namely Ethanol Expansion Programme (EEP) and ecoENERGY, for the biofuel program, with a budget of $78 million and $1.5 billion, respectively. In addition, the government reportedly funded the foundation of Sustainable Development Technology, Canada. This foundation provides non-repayable funds during the pre-commercial phase for novel technologies and processes. Also, 500 million dollars of funds were sanctioned under the next-generation biofuels fund for running private research centers. Similarly, BioFuelNet Canada, a research initiative was funded by Canada’s Networks of Centers of Excellence. The program focused on Canadian forest services, Transport Canada, and Agriculture Canada [21,22]. Likewise, the government of Russia invested 134 million USD for renewable energy production until 2030; while India invested 30 million USD in cellulosic-based bioethanol production [23,24]. Moreover, Governments, across the globe, are providing subsidies or rebates to co-op with low-priced gasoline or petroleum in fuel markets. For instance, in China, the government is providing a subsidy of 0.07 USD per liter of ethanol produced from cassava or sweet sorghum. Likewise, in South African, the government has decided to provide a 50% rebate on fuel levy for biodiesel producers [23]. In addition, several countries such as Russia, India, and Nigeria have allowed foreign investors to invest money for clean fuel production (Table 2).

**Table 2.** A snapshot of various biofuel policies in different parts of the continent [17,23,38–41]

Continent	Biofuel policies	Economics	Deliberation
Asia			
China	11 Provinces has implemented the 10% blending of bioethanol Biodiesel blending is varying from 2% to 5% Government provides the excise tax exemption for biodiesel produced from non-food crops or waste oil while no excise tax exemption for food crop-based bioethanol Tax rebates also apply on exported ethanol and biodiesel	The government provides a subsidy of 0.07 USD* per liter of ethanol produced from sweet sorghum or cassava No subsidies have been provided for biodiesel consumers and producers	China is the world’s fourth-largest bioethanol consumer and producer. Lowest official ethanol mandate. Biodiesel market cover 0.2% of total penetration and is still not expected to increase
Russia	Law of ‘Regulating the trade and production of bioethanol’ has been signed to open the Russian biofuels market High fuel excise tax on bioethanol	Investment of 134 USD for renewable energy production till 2030 ‘Russian Sustainable Energy Finance Program’ is a subsidy initiative to improve the capital flow for infrastructure, foreign investment and to cover financial losses because of low-priced fossil fuel	Biofuel projects in Russia are either supported by regional government or international investors Only two plants are producing ethanol from non-edible material Regulatory framework in Russia lacks to stimulate the biofuel production
India	Government proposed blending of 5–20% No excise tax reductions for biodiesel and bioethanol Import of biofuel are banned however import on feedstock required for biofuel production is permitted No mandate implementation of biodiesel and bioethanol in transportation sector	More than 30 USD million investment in cellulosic-based ethanol production Permission to foreign investment in biofuel technologies	Biofuels market in India nascent and have high GST tax The biofuel sector share 1.2% of total in transport sector The current advanced biofuel production is 1.75 million liters per year
Japan	Mandatory implementation of 500 million liter biofuels Zero delivery tax on 100% biodiesel Zero tariffs on bio-ethyl tert-butyl ether Tax incentives on bioethanol consumption	Ministry of Agriculture, Forestry and Fisheries has provided financial assistant and tax breaks to produce biofuel under ‘Basic Law for Promoting Biomass Utilization’	Limited bioethanol production and imported largely from Brazil Only 0.04% covers the transportation sector by biodiesel
Africa			
South Africa	Biofuel blending mandate of 10% and 5% for bioethanol and biodiesel Biodiesel producers are entitled of 50% rebate on fuel levy Excise exemption for bioethanol	Rebate of 50% on fuel levy for biodiesel producers	Plan to put quota for small-scale farmers to provide 25% feedstocks to biodiesel producers No subsidies and policies are in effect for biofuel commercialization
Nigeria	Bioethanol and biodiesel mandate blend of 10% and 20%, respectively Under national policy, foreign investors are allowed to contribute money for biofuel development	More than 414.7 million USD are spent to import bioethanol for industrial use UK-based group invested $340 million to develop bioethanol plant in 2016	Current production of bioethanol only accounts for 3% of total ethanol consumed Allowing the private sector will increase the number of investors in biofuel projects
Oceania			
Australia	Biodiesel and bioethanol blending varies from 5% to 0.5% and 6% to 4% in New South Wales and Queensland, respectively Grant schemes for biofuel production No nation-wide target for biofuel usage	Government grants and rebates on expenses and commercialization 1 billion dollars bioeconomy development in Queensland 10 billion dollars has been sanctioned to facilitate the clean energy sector 200 million dollars bioenergy funds	Few pilot plant has been developed for advanced biofuel production The current blending mandate in New South Wales is ineffective due to the lack of feedstock supply The benefits of bioethanol have been shown through advertisements in Queensland
New Zealand	Excise exemption on bioethanol while no exemption on biodiesel usage No biofuel blending mandate in transportation sector	Grant of 42.5 cents per liters for biodiesel production	No government policies for production of advanced biofuels Intermittent production of bioethanol or biodiesel due to lack of implementation mandates
Europe			
Germany	No tax exemption for bioethanol, hydrotreated vegetable oil and biodiesel The fuel tax of €0.0139 on biomethane and compressed natural gas 6.25% biofuel’s blending mandate	‘Energy transition in the transport sector’ is the initiative for advanced biofuel and conventional biofuel development.	‘German Mobility and Fuel Strategy’ program has implemented the use of renewable kerosene and its blends at Leipzig Airport. It resulted in less emission (30–60%) of particulates and carbon dioxide The use of bioethanol has increased to ~17% Several advanced biofuels projects are running in pilot scale
France	Incentives are introduced as well as subsidy for ethanol blend of more than 85% Biodiesel blending mandate is upto 8% Biofuel generated from first-generation should not be more than 7% of transport fuels Target to reduce the 30% fossil fuel-based energy consumption in the transport sector by 2050 has been set-up	Investment of 235.91 million USD to produce biodiesel from used cooking oil	Multiple advanced biofuel producing plants are under process such as FUTUROL, BioTfueL, and La Mede.
Sweden	Mandatory blending of bioethanol and biodiesel till 5% All the biofuels are fully tax exempted Pump law: Retailers with more than 1500 m^3^ per month fuel turnover should offer a biofuel blend of 50% or more Vehicles emitting more than 95 g CO_2_ per Km will be penalized up to 60,000 Swedish Krona	Government subsidies on commercial plants, pilot plants and for the programmes aims to reduce carbon emission are available	The consumption and production of biodiesel has increased to 37% and 32%, respectively More than 85% biofuels need to be imported to meet consumption demand 20% of transport markets covered by biofuels
Netherland	1.0% advanced biofuels, 17% biodiesel and bioethanol mandatory usage	Funds for the development of biofuel-based pumps	Most subsidies are for higher biofuel bend such as 85% for bioethanol and 30% for biodiesel Consumption of biodiesel and bioethanol has increased to 90% and 42%, respectively. Production has increased with a rat of 37% and 8% for bioethanol and biodiesel, respectively
Denmark	Carbon dioxide excise exemptions for biofuels Mandatory biofuel blending of 5.57%	Funds of 16 million USD for new biorefinery development 3.1 USD million has been approved for advanced biofuel production	No bioethanol production The funding programs are available for research and development but no specific programs for biofuel development
North America			
Canada	Blending of biodiesel and bioethanol varies from 2% to 4% and 5% to 8.5%, respectively, in different provinces Established the clean fuel standards to reduce the greenhouse emission till 2030 Provincial low carbon fuel standard and federal carbon pricing	Federal government programs and initiatives to increase biofuel production and commercialization are available 1.58 billion USD low carbon economy fund to reduce the greenhouse emission and increase clean fuel production	Canada requires to import the biofuel from US to meet the increasing demand Three commercial advanced biofuel producing plants are available Biodiesel exported to US is entitled to blender tax credits
The United States of America	Blender tax credits renewable diesel California’s low carbon fuel standard Aims to increase the biofuel production to 36 billion gallons per year by the end of 2022	Loan programs provide the risk management related to the scale of commercial projects The government provides wide range of programs to scale-up the biofuels and development of logistics and supply chain	Biofuel mandate in US has increased the consumption of biodiesel and bioethanol World largest bioethanol production with 58% of worlds ethanol production Numerous pilot plant, commercially advanced biofuel production plants runs in US
South America			
Brazil	Blending mandate up to 10% and 27% of biodiesel and bioethanol, respectively 14% import tariff on biodiesel Tax incentives and exemptions are available for biofuel producers, consumers and blenders Development of RenovaBio: a low carbon fuel standard policy	Incentives for feedstock developers such as sugarcane Credits for bioenergy industries and funds for the development of logistics, enhanced ethanol production and feedstock transportation	Two commercial cellulosic-based bioethanol production plant Biodiesel and bioethanol consumption has been increased by 18% and 3% per year, respectively

Additionally, each country is facing different problems associated with clean fuel production. For instance, in China, the biodiesel market, which is only 0.2% of the total transportation system, is not expected to increase due to the fact that no subsidy is being provided for biodiesel consumers as well as producers. Similarly, New South Wales is unable to follow the biofuel blending mandate laid by the Australian government due to the lack of feedstock supply. On the other hand, the lack of biofuel mandates has led to sporadic production of biofuels in New Zealand [23]. To fight the limited bioethanol production, Japan generally imports bioethanol from Brazil, while Canada imports biodiesel from the US. Hence, it is highly necessary that each country implement or mend the biofuel mandates to co-up with market prices, increase biofuel production, and decrease GHG emissions.

Regardless, the vast amount of resources spent on research on a renewable substrate such as lignocellulosic biomass, only a few of the lignocellulosic biomass-based biofuel producing commercial plants are functional, some of them are under construction in Asia, Europe, and America [25]. This is because lignocellulosic biomass is a complex structure, hence it requires the pretreatment to increase the accessibility of sugars, which corresponds to around 40% of total sugar production cost resulting in scale-up issues and plant shutdown [26,27]. For instance, KiOR Inc., which was based in the U.S. faced problems in ramping up the thermal conversion of biomass due to structural design problems which led to its operational shutdown [28]. On the other hand, Biochemtex/Beta renewable had faced bankruptcy due to pretreatment difficulties and lignocellulosic biomass complexity [27] and was recently acquired by Eni in 2018. The company is currently planning to ramp up the Beta Renewables Proesa technology of converting biomass into second-generation sugars. Moreover, major efforts are underway to find out the optimum pretreatment method in terms of enhanced biomass disintegration, low chemical requirement, life cycle assessment and techno-economic feasibility.

So far, numerous techno-economic analyses on biofuel production have been performed. It acts as a connecting bridge between small scale and commercial-scale production. In general, techno-economic analysis has been performed using different substrates (e.g. agriculture, forestry, sea and industrial residues), several pretreatment methods (pyrolysis, thermochemical, steam explosion, ball milling and micronizing), and products such as jet fuel, bioethanol, biobutanol, bioethanol, biodiesel, sugar hydrolyzate and renewable gasoline [10,29–32]. On the contrary, techno-economic analysis is generally performed assuming the ideal conditions such as using one type of substrate, biomass availability either inside the plant or in its vicinity hence eradicating the transport cost, discounts, supplementations and incentives. On the other hand, failing the single assumption might lead to an increase in biofuel price or a plant shutdown. For instance, a change in the type of substrate could increase the structure complexity such as in hardwood, softwood, corn stover or miscanthus, resulting in high energy requirement than anticipated, which will ultimately add the cost to the final price. Similarly, yearly substrate availability, transportation, processing and storage play a key role in production cost. Likewise, most of the studies suggested the use of own warehouse while subcontracting the warehouse could be an alternative and cost-effective option. Therefore, a deeper understanding of logistics, operation facility, process gaps, risk assessment on each parameter would be required to mitigate the process and capital-related setbacks.

Nonetheless, the final cost of biofuel production seems to exceed the market cost of fuels ($3/gallon), which are the key challenges in scaling up the projects [5]. To confront this problem, co-production of high-value compounds such as phenolic derivatives, essential fatty acids, enzymes or green chemical products such as furans, lignin fractions could be an alternative option. However, it will require the extra chemicals, maintenance or downstream cost hence raising the life cycle assessment (LCA) methodology challenges. For instance, Cai et al. [33] performed the LCA study of co-produced adipic acid and succinic acid during renewable biodiesel production. During this condition, additional chemicals and energy would be needed to produce the co-products. Each product conversion is entirely dependent on the energy applied, the substrate used and the chemical provided in the operation of each unit. Nevertheless, the market of co-products for biorefineries requires further studies and exploration to draw a conclusion. Furthermore, as the technology matures, LCA results, product yield, and economic analysis will be important for further biorefinery development and expansion. Hence, this is time to re-contemplate and modify the current strategies on renewable biorefinery development.

The following options could be considered for further analysis:

- Scale-up of lignocellulosic biomass relies on its type, complexity and pretreatment. Hence, a deeper understanding of the type of pretreatment effect on biomass complexity and employment of cost-effective strategy is required.
- Implementation of zero waste production and proper handling of waste produced after pretreatment of biomass.
- Detailed evaluation of different types of feedstocks and their potential effect on scale-up. Modification in techno-economic software to extrapolate the economic performance among different feedstocks.
- Production of high-value compounds as co-products should require further exploration as their production might saturate the market.

## 5. Future perspective

Over the last two decades, biofuel industries have seen a lot of ups and downs. Several advances in biofuel production have been introduced over time, such as new and improved catalysis, advanced pre-treatment techniques, genetic engineering of microorganisms, step integration, processes modification, co-production strategy, and increase in substrate diversity. Governments have initiated several funding programs to push forward renewable biofuel production and started numerous campaigns to increase biofuel consumption and promote the reduction of GHG emissions [34]. However, the critical question that remains unanswered is, how can advanced biofuel and biorefinery be adopted in the mainstream? One approach could be by developing and expanding technologies, increasing feedstock availability and promoting policy implementation and foreign collaborations.

Nevertheless, COVID-19 has brought unforeseen consequences in terms of lowest economic growth, unemployment, and business shutdowns. Most significant ones, with respect to the bioenergy sector including a considerable reduction in total energy demand, transport fuels and biofuels. For instance, as per the rate of change of energy demand graph, published by the International Energy Agency (IEA), global energy demand has decreased by 10% in 2020 which has not been seen in the last 70 years [35]. Likewise, a 13% reduction in biofuel usage has been recorded in 2020 compared to the past 10 years. Additionally, the forecasted value of annual biofuel production to meet the GHG reduction had dropped from 7% to 1.9% [36]. Moreover, carbon dioxide (CO_2_) emission has also fallen over the last year, i.e. 4.5% from oil, 8% from coal and 2.3% from natural gas, which corresponds to 2.5 gigatons of CO_2_. Although, the reduction in carbon dioxide was desired but not due to severe economic disruption and strict lockdown worldwide. In addition, lockdown during the pandemic also resulted in the sudden stoppage of the supply chain system of several industries including the bioenergy resources other than essential services. This is another integral factor in the successful deployment of products in the market [5]. This could either boost the product flow or could temper the flow based on adaption to sudden market change. Moreover, the exiting policies lack in recognizing the key role of bioenergy sector and its supply chain, as it should be categorized under essential services not just during the existing pandemic but as a general rule for the future generation. Also, supply chain management of bioenergy industries should be completely digitized, which would not only allow to monitor the flow of resources through online tracking system but also could provide the opportunity to divert the hampered system toward transportation of other essential services. Moreover, nations across the world are receiving pakages and funds to support companies and communities, while it is necessary to have bioenergy as one of the category or else the progress made over the decade in bioenergy sector might lose.

So far, Canada’s CFS policy to reduce GHG emissions has gained worldwide attention. The CFS with provincial policies and carbon tax schemes are expected to escalate the use of biofuels to drive the change in the vehicle fleet and fuel market of Canada. However, Canada does not have a tax credit scheme on the blending of biofuels. For instance, the US provides the biodiesel blender credits of US $1/3.7 liters of biodiesel used for the blending process which resulted in the world’s maximum biodiesel production of 606 million liters per year in the US. In addition, Canadian exporters are also eligible for biodiesel blender tax credits. In this sense, 80–90% of biodiesel produced in Canada is generally exported to the US to get these tax credits as well as Renewable identification numbers (RIN) [37]. Canadian biodiesel companies reportedly obtain 70% of the blender credits which they use to import double the amount of biodiesel exported to meet the biodiesel demand in Canada. This could be due to the lack of biodiesel production infrastructure in comparison to demand in Canada hence it is necessary to import a significant amount of biodiesel from a country like the USA.

**Figure 3.**
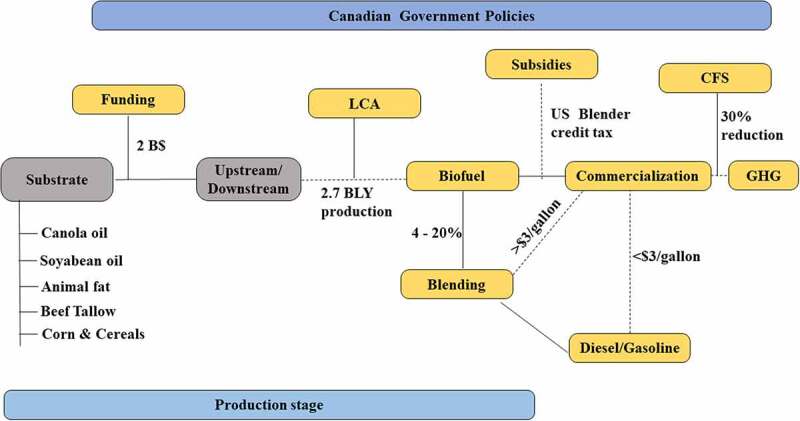
Summary of Canadian government policies on biofuel production, commercialization and GHG emission reduction: where, B$: billion dollars; BLY: billion liters per year; LCA: life cycle assessment; CFS: clean fuel standards; GHG: greenhouse gas

Figure 3 displays the Canadian government policies and their impact on different stages in biorefinery. It is evident that economics plays a crucial role in sustaining the biofuel industry. Biorefinery remains a key concept in improving the economics of the biofuel industry. For instance, in Canada, forestry and pulp-paper industries are trying to integrate waste residues with biochemicals and biofuels production. The waste from forestry and paper-pulp industries includes lignin, extractives, cellulose, hemicellulose, and wastewater, which can be used for value-addition products if harnessed wisely. For instance, Alberta Pacific Forest Industries currently produce 4000 tonnes of biomethanol required for chlorine dioxide production via stripping from the waste gas stream. Likewise, Domtar corporation produces nanocrystalline cellulose using a kraft pulp waste stream [34].

## 6. Conclusion

The present study provides critical information on perspective, regulations and the current status of biofuel in Canada while comparing it with international policies. In a nutshell, development and innovation funding will be beneficial for translating concepts to biofuels and improving yields. Nevertheless, the renewable biofuel industry is still under exploration and requires further studies to optimize biofuel production using feedstock other than crops. The advanced biofuel production in Canada is encouraging and extensive. With the combined efforts of industry, government and academia, significant progress in achieving the GHG reduction target is indeed possible.
